# Special characteristics of human astrocytes and their roles in brain homeostasis

**DOI:** 10.1016/j.nsa.2026.107000

**Published:** 2026-04-16

**Authors:** Ilari Kousa, Karo Talvio, Ulla-Kaisa Peteri, Maija L. Castrén

**Affiliations:** Department of Physiology, Faculty of Medicine, University of Helsinki, Haartmaninkatu 8, 00290, Helsinki, Finland

**Keywords:** Human, Astrocytes, iPSC, Organoids

## Abstract

Human astrocytes differ morphologically, functionally, and in their gene expression patterns from murine astrocytes. The astrocytic heterogeneity and complexity observed in the mouse brain are multiplied in humans, evident in primate-specific astrocyte populations and region-specific subtypes reflecting diverse molecular programs and environmental factors in the human brain. Astrocytes also contribute to the evolutionary processes associated with an increase in brain size and enhancing cognitive function. In this review, astrocyte differentiation and function are described to frame the processes that make human astrocytes specific, and whose disturbances can lead to brain pathology, as revealed by rapidly-evolving stem cell-based technology employed in human astrocyte studies.

## 1 What makes human astrocytes special?

Astrocytes are an abundant glial cell population in the central nervous system (CNS) (Azevedo et al., 2009). Recent research has highlighted the functional importance of astrocytes and variation across species. Astrocytes represent a morphologically and functionally heterogeneous group of cells with brain region- and species-specific properties. Throughout evolution, the increased number and structural complexity of astrocytes have played a role in supporting enhanced cognitive capacities in humans (Ciuba et al., 2025). Studies have demonstrated that when human astrocyte progenitors are transplanted into the mouse brain, they can lead to improvements in learning and cognitive function (Han et al., 2013).

Human astrocytes exhibit a higher degree of heterogeneity and distinct features that set them apart from murine astrocytes in morphology, gene expression patterns, and function (Chandrasekaran et al., 2016). Human astrocytes are larger than their murine counterparts (Oberheim et al., 2009). The astrocyte-to-neuron ratio reflects organismal complexity: while the mouse cortex has an average ratio of 1:3, the human cortex displays a ratio of 1:1.4 (Nedergaard et al., 2003). Bidirectional interactions between astrocytes and neurons contribute to the complexity of mechanisms governing information processing and plasticity within the CNS. Astrocytes are characterized by primary processes that extend from the cell soma and branch into higher-order processes, terminating in leaflets and endfeet. Leaflets, known as perisynaptic astrocytic processes (PAPs) interact with synaptic structures. By ensheathing synapses, they act as a barrier for limiting spillover of neurotransmitters and ions, and support synaptic homeostasis (Allen and Eroglu, 2017). In synapses, a single protoplasmic astrocyte in the mouse cortex contacts, on average, 20,000–120,000 synapses, whereas in the human cortex the corresponding number is 270,000–2,000,000 (Oberheim et al., 2009). The capacity of astrocytes to perform versatile functions depends on their dynamic structure (Semyanov and Verkhratsky, 2021). Endfeet, or perivascular processes (PVPs), are specialized structures that anchor astrocytes to cerebral blood vessels (Semyanov and Verkhratsky, 2021).

Transcriptomic analyses of astrocytes underscore interspecies differences by showing that only ∼30% of human astrocyte-enriched genes are enriched in mice (Zhang et al., 2016). Expression of genes involved in defence mechanisms and metabolism points significant differences between mouse and human astrocytes reflecting diversity in astrocytic responses to hypoxia and demonstrate a greater susceptibility of human astrocytes to oxidative stress (Li et al., 2021a). Additionally, human astrocytes possess a heightened ability to activate the antigen pathway when exposed inflammation. Acute or chronic adverse conditions induce morphological and molecular changes in astrocytes, resulting in so-called ‘reactive states’. These states include many reactive astrocyte subtypes, some of which may be specific to humans (Escartin et al., 2021).

Although each astrocyte predominantly occupies a distinct territorial domain that does not overlap with others, they remain highly interconnected. Extensive gap-junctional coupling facilitates interconnection, allowing astrocytes to form syncytial networks, which enable the direct exchange of ions, nutrients, and signalling molecules as well as propagation of intercellular Ca^2+^ waves (Pannasch et al., 2012). Notably, human protoplasmic astrocytes have been shown to propagate calcium waves in cortical slices speeds approximately four times faster than those in rodent astrocytes (Oberheim et al., 2009). Human astrocytes also acquire glutamate responsiveness later during gestation than mouse astrocytes (Zhang et al., 2016). The species-specific features of astrocytes significantly contribute to the uniqueness of the human brain particularly in relation to several plasticity processes modulated by astrocytes. These processes encompass the formation, structural shaping, and pruning of synapses in addition to removal of excess neurotransmitters to mitigate excitotoxicity (Allen et al., 2012; Molofsky et al., 2012; Rothstein et al., 1996; Pfrieger and Barres, 1997; Ullian et al., 2001; Allen and Lyons, 2018; Chung et al., 2015; Cinquina et al., 2025). The present review highlights unique characteristics of human astrocyte development resulting in remarkable diversity, distinct subtypes, and specific morphological and functional properties of human astrocytes, which may be seen in human cell models, providing the potential for the study of human diseases.

## 2 Development of astrocytes

**Fig. 1:**
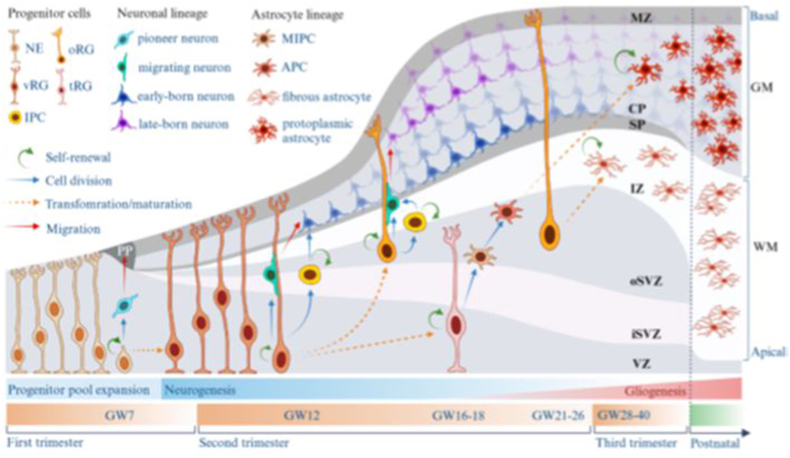
Early cortical development in the human cortex. At the onset of the cortical development, neuroepithelial (NE) cells divide symmetrically to expand the progenitor pool. Generation of the first neurons, pioneer neurons, leads to the formation of the preplate (PP) and, subsequently, the marginal zone (MZ), cortical plate (CP), and subplate (SP). Emergence of neurons also causes NE cells to transform into ventricular radial glia (vRG) around the end of the first trimester. vRG generate neurons either directly or via IPCs and immature neurons migrate to the cortex along vRG processes. Neurons occupy cortical layers in an inside-out manner which leads to the formation of the six cortical layers. vRG transform into basal/outer radial glia (oRG) and truncated radial glia (RG) attached to the basal and apical surfaces, respectively. Similarly to vRG, oRG generate neurons either directly or indirectly via intermediate progenitor cells (IPCs). At the onset of gliogenesis, tRG generate MIPCs that in turn give rise to APCs. APCs migrate to CP where they mature into protoplasmic astrocytes of grey matter. White matter fibrous astrocytes are produced by oRG. Generated astrocytes proliferate locally in the cortex and acquire their final properties and morphology postnatally. Abbreviations: CP, cortical plate; GM, grey matter; GW gestational week; IPC, intermediate progenitor; iSVZ inner subventicular zone; IZ, intermediate zone; MZ, marginal zone; NE, neuroepithelial cell; oRG outer radial glia; oSVZ, outer subventricular zone; tRG, truncated radial glia; VZ, ventricular zone; WM, white matter. Created in BioRender. Peteri, U. (2025) https://BioRender.com/h80e457.

The generation of glial cells follows neurogenesis. In the human cortex, the first astrocyte progenitors appear in mid-gestation, and mature astrocytes begin to emerge from approximately gestational week (GW) 20 onward (Roessmann and Gambetti, 1986). Most astrocytes are generated between GW23–26, although astrocyte maturation continues postnatally (Roessmann and Gambetti, 1986; Falcone et al., 2021; Zhong et al., 2018). Fig. 1 depicts the differentiation of neuronal cells during early development of the human cerebral cortex.

### 2.1 Origin of astrocytes from RG and gliogenesis

Radial glial (RG) are the first cell type of astroglial lineage (Kriegstein and Alvarez-Buylla, 2009). They emerge from the neuroepithelial cells (NEs) by the anterior part of the neural tube around the end of the first trimester of human pregnancy (Subramanian et al., 2017). In mouse, NEs lining the cerebral ventricles begin to transform into RG around E9-10 (Kriegstein and Alvarez-Buylla, 2009). The ventricular RG (vRG; also referred to as apical RG) contact both the ventricle and the pia. Initially neurogenic RG give rise to two lineages, which contribute to neocortical astrocyte diversity (Zhou et al., 2025). The subventricular zone (SVZ) is formed around post-conceptional week (pcw) 12 of human development as a distinct secondary proliferative zone between the highly proliferative ventricular zone (VZ) and the non-proliferative intermediate zone (IZ) (Sidman and Rakic, 1973). vRG give rise to basal/outer progenitors (oRG) in the outer SVZ (oSVZ), where they lose their apical contacts while retaining their basal processes (Ortega et al., 2018). Around pcw 14-14.5 in humans, vRG transform into truncated RG (tRG) and adopt a specific molecular program (Keefe et al., 2025; Nowakowski et al., 2016).

The oSVZ with intermediate glial progenitors (Li et al., 2021b; Weng et al., 2019) accounts for multiple neuronal and glial cell types during intrauterine development. It is larger in humans than in other species and the extended proliferation of oRGs contributes to the remarkable cortical expansion and gyration in humans and other gyrencephalic mammals (Hansen et al., 2010; Kriegstein et al., 2006; Shitamukai et al., 2011; Vaid et al., 2018). In the developing mouse brain, oRGs represent a minor cell population (Shitamukai et al., 2011; Vaid et al., 2018). Fibers of tRG do not extend beyond the SVZ and they often terminate on the capillaries in the inner SVZ (iSVZ) and the oSVZ (Nowakowski et al., 2016). Both oRG and tRG can self-renew and generate all cell types with potential clonal coupling (Keefe et al., 2025). As the later stage progenitors, tRGs give rise to ependymal cells and astrocytes (Bilgic et al., 2023), and they remain neurogenic in the late second trimester in the developing human cortex (Keefe et al., 2025).

Generation of cortical astrocytes has been divided into three phases: early gliogenesis, which produces gliogenic precursors; intermediate gliogenesis, which generates white matter fibrous astrocytes; and late gliogenesis, which produces grey matter protoplasmic astrocytes (Marin-Padilla, 1995). However, mapping the developmental trajectory of human astrocyte differentiation defined three modules with additional transitory early/middle and middle/late signatures (Sojka et al., 2025). Transcriptomic signatures of early astrocyte stages (D80–D250) associated with a proliferative (MKI67, TOP2A, ID3, and NES) cell state and physiological properties such as early response to environmental cues and cell motility. The middle stage of astrocyte maturation is enriched by glial differentiation genes, including the fate determining transcription factor genes such as SOX9, OLIG1/2, LHX2, POU3F3 (BRN2), EMX1, and SOX2. Later maturation phases included quiescent mature genes (GJA1, AQP4, and ALDH1L1) and those associated with CNS vascular formation, ion homeostasis, astrocyte-mediated synapse organization, and metabolic support.

During early gliogenesis, tRG-derived subpopulation of basal multipotent intermediate progenitors (bMIPCs) expresses epidermal growth factor receptor (EGFR), ASCL1, OLIG1, and OLIG2 similarly in mouse and human (Li et al., 2021b). The bMIPCs undergo several rounds of mitosis to generate oligodendrocyte progenitor cells (OPCs) and astrocyte progenitor cells (APCs), and to give rise to oligodendrocytes, astrocytes, and olfactory bulb interneurons (Zhou et al., 2025; Yang et al., 2022).

In the early embryogenesis, astrogliogenesis in the human cortex is based on the production of intermediate glial progenitors through asymmetric divisions of RG. These precursors migrate to colonize their final positions, proliferate, and transform into astrocytes. Postnatally astrocytes are also generated directly from RG, which at around birth lose their apical processes and transform into protoplasmic astrocytes (deAzevedo et al., 2003; Voigt, 1989). In the developing cortex, astrocytes and their precursors may coexist at multiple developmental stages. The cell type-specific mutagenesis and fate-mapping analyses in mouse have provided evidence that the two classical subtypes of astrocytes, protoplasmic and fibrous astrocytes, are generated in different ways (Cai et al., 2007). Gray matter protoplasmic astrocytes are generated mainly by tRG-derived progenitors, and to a lesser extent by NG2 cells, which primarily are oligodendrocyte progenitors and shown to generate local ventral forebrain protoplasmic astrocytes (Marin-Padilla, 1995; Nishiyama et al., 2016). Because of their dual origin, astrocytes display a bilaminar pattern of localization during development (deAzevedo et al., 2003).

Unlike neurons, immature astrocytes do not populate the cortex in a strictly laminar manner, although they can migrate along RG fibers (Yang et al., 2022). Astrocyte progenitors are shown to migrate in a non-ordered manner combined with restrictions caused by region-specific neuron-astrocyte interactions and intrinsic factors (Tsai et al., 2012). Transplantation studies in rats have shown that the degree of migration is developmental stage-dependent; at younger ages astrocytes migrate relatively freely, whereas in older animals increasing region-specific preferences exist (Hatton et al., 1993). Once in their final positions, astrocytes continue to proliferate locally and gradually acquire their mature morphology and properties, which are shaped by their microenvironment (Clavreul et al., 2019). Final astrocyte maturation requires neuronal input and occurs in parallel with the establishment of functional neural circuits (Stogsdill et al., 2017). The developmental programs and extrinsic stimuli lead to subsets of astrocytes with regional differences in functional, morphological, and transcriptional properties. Furthermore, even mature astrocytes retain a degree of plasticity, and they can act as progenitors in the adult brain (Doetsch et al., 1999; Patten et al., 2003).

### 2.2 Molecular pathways driving astrocyte differentiation

The development of cortical structures requires tightly coordinated temporal and spatial cues. The gliogenic switch, the shift in progenitor differentiation from neuronal to glial lineages coordinates timing of astroglia differentiation. The principal pathways that govern astrocyte differentiation have been explored exclusively in rodent models. Although similarly detailed information regarding the regulatory mechanisms is lacking, fundamental primary mechanisms appear mainly conserved and the molecular mechanisms are effectively engaged in human astrocyte differentiation from pluripotent stem cells (Doetsch, 2003; Tcw et al., 2017), During the neuronogenic period, differentiation of glia is suppressed by neurogenic basic hex-loop-helix (bHLH) proneural factors, including neurogenin 1. When an appropriate number of neurons is generated, multiple extrinsic and intrinsic mechanisms act in concert to induce gliogenesis. A crucial role of cytokines has been demonstrated in vivo by showing that newly born mouse neurons secrete cytokines such as leukemia inhibitory factor (LIF), cardiotrophin-1 (CT-1), and ciliary neurotrophic factor (CNTF) (Barnabe-Heider et al., 2005), which all bind a receptor complex comprising the LIF receptor (LIFR) and its co-receptor glycoprotein-130 (gp130), required for astrogenesis (Ware et al., 1995). CT-1 is a key gliogenic ligand and its ablation of neuron-derived CT-1 completely blocks neurogenic gliogenic transition in cultured cortical precursors (Barnabe-Heider et al., 2005). When the ligand is CNTF, the ciliary neurotrophic factor receptor α (CNTFRα) also associates with the receptor complex (Bonni et al., 1997). Receptor assembly activates Janus kinases (JAKs), which phosphorylate STAT3. Phosphorylated STAT3 dimerizes and translocates to the nucleus to drive transcription of astrocyte genes, including glial fibrillary acidic protein (GFAP) (Bonni et al., 1997; Koblar et al., 1998). This signalling pathway is known as the Janus kinase/signal transducer and activator of transcription pathway (JAK/STAT), which cooperates with other transcription factors to promote gliogenesis in the mammalian brain.

Rodent studies have shown that neural progenitor stemness is maintained by growth factors such as fibroblast growth factor 2 (FGF2) and neuregulin-1 (NRG1). NRG1 activates erythroblastic leukemia viral oncogene homologue (ERBB) family receptors, which repress astrocyte genes in coordination with the nuclear receptor co-repressor N-CoR (Fox and Kornblum, 2005; Sardi et al., 2006). The transition of RG to astrocytes is triggered by the gradual suppression of NRG1 expression (Hirabayashi et al., 2009). Notch signalling helps maintain RG stemness and prevents premature differentiation at early stages (Campos et al., 2001; Yoon et al., 2008). It primes RG for astrocyte production through demethylation of astrocyte-specific genes and by enhancing their responsiveness to cytokines at later stages (Chambers et al., 2001; Namihira et al., 2009). Notch upregulates the transcription factors Sox9 (SRY-box transcription factor 9) and NFIA (nuclear factor I A) (Namihira et al., 2009; Martini et al., 2013), which act cooperatively to induce astrocytic gene transcription (Kang et al., 2008). Notch and JAK-STAT pathways interact and are influenced by epidermal growth factor (EGF) (Kamakura et al., 2004) and bone morphogenetic protein 2 (BMP2) signalling during astrocyte differentiation in a stage-dependent manner (Ochiai et al., 2001). Early in development, BMP promotes apoptosis and inhibits proliferation; later, it supports differentiation - initially toward neuronal fates and subsequently toward astrocytes (Mabie et al., 1999).

### 2.3 Astrocyte heterogeneity and human-specific astrocytes in the cortex

Astrocytes have traditionally been divided based on morphology into protoplasmic astrocytes in gray matter, with a branched stellate morphology, and fibrous astrocytes in white matter, where they exhibit a more elongated morphology and fewer branches (Miller and Raff, 1984; Kettenmann and Verkhratsky, 2008). Fibrous astrocytes express higher levels of GFAP than protoplasmic astrocytes (Marin-Padilla, 1995; Miller and Raff, 1984), and they are generally considered to provide metabolic support rather than directly participate in information processing (Oberheim et al., 2006). Dense bulbous protoplasmic astrocytes emerge early (Keefe et al., 2025), coincident with GM vascularization (Marin-Padilla, 1995). They are closely associated with synapses and implicated in the regulation of synapse formation and function (Chung et al., 2015). Both human protoplasmic and fibroblastic astrocytes are larger than their murine counterparts (Oberheim et al., 2009).

Interlaminar astrocytes (ILAs) are morphologically distinct astrocytes in the human brain (Oberheim et al., 2006; Ciani and Falcone, 2024). They were identified as GFAP-positive astrocytes in the cortical layer I contributing to the outer glial limiting membrane (Colombo et al., 1995). ILAs have more recently been found in all mammals, but with human-specific morphological differences. Between species shared properties of ILAs include a cell body close to the pia mater and at least one process traveling perpendicular to the pia (Falcone et al., 2019). In humans, ILAs display higher density and complexity. Their long inter-laminar processes, which extend to lamina IV-V and replicate the columnar organization of neurons, form a dense fiber network and promote radial interaction between different layers. Human ILAs are generated relatively late prenatally and mature postnatally (Marin-Padilla, 1995). A unique miRNA profile of ILAs likely reflects the status of a specialized astrocyte population (Rao et al., 2016). They are hypothesized to support larger brains by modulating neuronal activity (e.g., propagating Ca^2+^ waves) and draining excess ions via long processes (Colombo and Reisin, 2004).

Varicose projection astrocytes (VPAs) have been previously considered specific to hominoids (Falcone et al., 2022). VPAs comprise a relatively small astrocyte population located in layers V–VI near WM. They possess short, spiny processes with regularly spaced varicosities and a few long processes that can invade neighbouring astrocyte domains and often contact vasculature (Oberheim et al., 2009). VPAs express astrocytic markers GFAP, AQP4, and S100B as ILAs. Notably, VPAs are not detected in all specimens within a species, suggesting that VPAs may represent a state responsive to external stimuli rather than a fixed cell type.

**Fig. 2:**
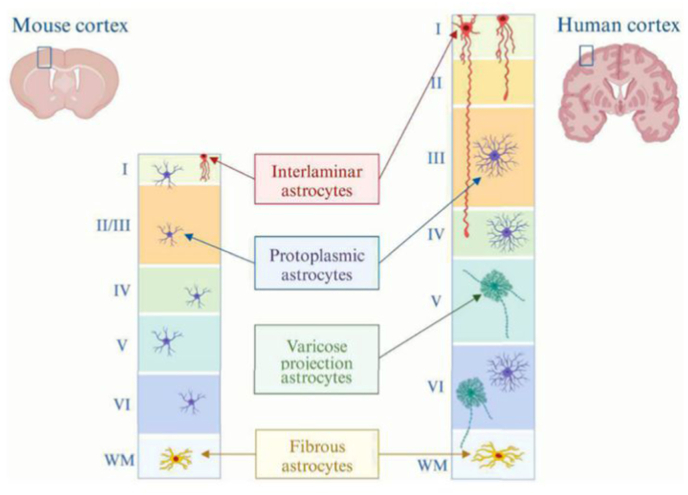
Astrocyte subpopulations with distinct morphology in the mouse and human cortex. Schematic representation illustrates the main types of cortical astrocytes in both the mouse and human brain. Interlaminar astrocytes (ILAs) are characterized by having their nuclei located in the cortical layer I. In human, ILAs send processes in the deeper layers while mouse brain contains rudimentary ILAs confined to the layer I. Protoplasmic astrocytes are the most abundant type of astrocytes in both mice and humans. Varicose projection astrocytes (VPAs) are specific to higher-order mammals and possess processes with evenly spaced varicosities. Fibrous astrocytes are found in the white matter of both mouse and human brains. Created in BioRender. Castren, M (2025) https://BioRender.com/5m0ttnn.

Morphological analysis enables the identification of a limited number of cortical astrocyte populations in the human brain (Fig. 2). However, morphology is not a reliable criterion for classifying astrocytes, nor is a single astrocyte marker study. For example, the commonly used marker GFAP is also expressed in human radial glial cells (Kadhim et al., 1988). Single cell transcriptional profiling has been used to spatially map neuronal cells in the CNS, providing a more specific, data-driven taxonomy of astrocytes in both the mouse and human brain (Siletti et al., 2023; Zeisel et al., 2018). Two types of astrocytes—telencephalic and non-telencephalic—express low or high levels of GFAP, a conserved feature in humans and mice. Single-cell/nuclei-RNA-sequencing (sc/sn-RNA-Seq) studies of postmortem human brain tissue have shown that telencephalic astrocytes include both protoplasmic and fibrous astrocyte subclusters, as well as ILAs (Zeisel et al., 2018). Distinct region-specific functional subtypes have been identified based on the developmental and anatomical compartments of astrocytes (Zeisel et al., 2018), indicating that human-specific developmental programs together with extrinsic factors contribute to the increased heterogeneity of human astrocytes.

## 3 Functions of astrocytes

Present ubiquitously in the brain, astrocytes regulate the formation and activity of individual synapses as well as neuronal networks. Their additional roles include providing metabolic support to neurons and various homeostatic functions in response to insults to the brain. Furthermore, through their role in the blood brain barrier (BBB), they are positioned to interact with peripheral signals and to control local blood circulation. Although these fundamental roles of astrocytes are conserved across species, distinct specializations enable them to support the functional demands of the human brain. Comparison of human and other primate astrocytes has revealed differently expressed genes to be enriched in synapse biology, metabolism, extracellular exosome, and nuclear factors (Ciuba et al., 2025). Additionally, emphasizing the importance of astrocytes to human pathology, genes related to neurological diseases, including intellectual disability, are downregulated in human astrocytes (Ciuba et al., 2025).

### 3.1 Developmental functions of astrocytes

The emergence of astrocytes coincides with brain vascularization in humans (Marin-Padilla, 1995), and based on mouse studies, contact with blood vessels is considered a hallmark of cortical astrocytes (Hösli et al., 2022). They contribute to the blood brain barrier (BBB) through their endfeet (Schiera et al., 2024), which are enriched in aquaporin-4 (AQP4) channels that mediate water transport (Haj-Yasein et al., 2011), regulate calcium signaling, and influence motility (Saadoun et al., 2005; Ciappelloni et al., 2019) in rodents. These vascular contacts also facilitate the regulation of cerebral blood flow (Abbott et al., 2006; Marina et al., 2020). Differential GFAP staining between mouse and human astrocytes indicates specialized vascular endfeet in human astrocytes (Oberheim et al., 2009), and human astrocytes have more AQP4 on their parenchymal membranes (Eidsvaag et al., 2017). However, the significance of these species differences remains to be studied.

Astrocytes secrete various factors that remodel the extracellular matrix (ECM) and regulate cell growth, migration, and adhesion. ECM remodelling is closely connected to synapse formation and maturation. These developmental mechanisms have been elucidated primarily in mice. Astrocytes use both secreted and membrane-associated proteins to promote synaptogenesis, synaptic maturation, and synapse pruning (Allen and Eroglu, 2017). Some factors are involved in synaptogenesis (Christopherson et al., 2005), while others strengthen and regulate the activity of pre-existing synapses (Allen et al., 2012). Among secreted molecules, proteoglycans and glycoproteins, such as tenascin (TNC), facilitate axonal growth and synaptic plasticity (Wiese et al., 2012). Thrombospondins 1 and 2 (TSP-1 and TSP-2), mainly expressed in immature astrocytes, induce postsynaptically silent synapses lacking AMPA receptors (Christopherson et al., 2005). In contrast, glypicans 4 and 6 (Gpc4 and Gpc6) promote the recruitment of AMPA receptor subunit GluA1 to neuronal membranes (Allen et al., 2012). Astrocytic secretion of high endothelial venule protein (hevin) promotes the formation of excitatory synapses, whereas secreted protein acidic and rich in cysteine (SPARC) is a negative regulator of synapse formation (Kucukdereli et al., 2011). Although these mechanisms have not been studied in human astrocytes, expression studies of TNC (Pena et al., 1999), TSP (Garcia et al., 2010), Hevin (Nunez-delMoral et al., 2021), and SPARC (Jones and Bouvier, 2014) suggest conservation across species.

Plasminogen activator (PA) system is involved in tissue repair in the CNS, and both tissue PA and urokinase PA are expressed in human astrocytes. They promote ECM degradation, axonal growth, neuronal migration, dendritic branching, and neuronal plasticity (Yepes, 2024; Peteri et al., 2021). Astrocytic PAs cleave pro-forms of growth factors such as hepatic growth factor (Naldini et al., 1992) and neurotrophic factors (Seidah et al., 1996) into their mature forms, which provides an additional astrocyte-mediated mechanism of plasticity regulation.

### 3.2 The tripartite synapse

Astrocytes modulate and coordinate signaling in neural networks. The larger size and faster calcium wave propagation facilitates the organization of larger astrocyte domains in humans (Oberheim et al., 2009), possibly supporting the coordinated functioning of a greater number of neurons. Astrocytic regulation of excitability by extracellular potassium levels has been demonstrated in both mice and humans (Bellot-Saez et al., 2018; Li et al., 2001; Xiao and Hu, 2014; Higashimori and Sontheimer, 2007).

Astrocytes partake in synaptic transmission through the metabolism of neurotransmitters (Andersen and Schousboe, 2023; Liu et al., 2022; Minelli et al., 1996; Pellerin and Magistretti, 1994) and the release of gliotransmitters regulated by calcium waves (Goenaga et al., 2023). Compared with other primates, human astrocytes express more EAAT2 (Munger et al., 2022), which may signify more active neurotransmitter metabolism in human astrocytes, leading to the accentuation of excitatory synaptic transmission and long-term depression (LTD) when human astrocytes are allografted into mice (Han et al., 2013). Human astrocytes acquire glutamate sensitivity later than mouse astrocytes (Zhang et al., 2016). Greater involvement of human astrocytes in neuroplasticity is also evident as increased dendritic spine turnover in a chimeric mouse model (Anding et al., 2025). How altered calcium dynamics influence gliotransmission by human astrocytes remains to be studied in detail.

### 3.3 Homeostasis

Astrocytes provide metabolic support to neurons. They take glucose from the blood via GLUT1 and store it in glycogen granules (Cali et al., 2016; Nguyen et al., 2021). For neuronal energy demands, astrocytes metabolize glucose into lactate, which they export for use by neurons, which can convert it back to pyruvate for oxidative metabolism (Pellerin and Magistretti, 1994; Kim et al., 2025; Dembitskaya et al., 2022). Astrocytes also uptake fatty acids through the BBB (Bernoud et al., 1998), regulate lipid availability in the brain (Mi et al., 2023; Chen et al., 2023), and produce cholesterol (Bloch et al., 1943; Genaro-Mattos et al., 2019; Bjorkhem and Meaney, 2004). However, mouse astrocytes express more metabolism-related genes, independent from the species of the neuronal context, and the basal respiration rate of human astrocytes is only half of that of mouse astrocytes (Li et al., 2021a). Compared with other primates, human astrocytes have larger GLUT1 vessels (Munger et al., 2022), and genes overexpressed in human astrocytes are enriched in metabolism, suggesting that certain metabolic changes are human specific. While human astrocytes may have reduced capacity to transport lactate, they express more PDHA1 (Pyruvate Dehydrogenase E1 subunit alpha 1), which converts pyruvate into acetyl-CoA, and less LDHB (lactate dehydrogenase B), which converts pyruvate into lactate (Zintel et al., 2024), suggesting that human astrocytes may be relatively more involved in lipid and neurotransmitter metabolism.

Astrocytes are immunologically active and exhibit responsivity and adaptation resulting from injury to the brain (Hol and Pekny, 2015). Reactivity leads to cellular hypertrophy and greater morphological complexity, facilitating glial scar formation and interactions that can regulate neuronal survival and death (Pekny et al., 1999; Wilhelmsson et al., 2004, 2006; Liddelow et al., 2017; Faulkner et al., 2004; Morizawa et al., 2017). Duality in astrocyte reactivity has been framed as neurotoxic and neuroprotective subtypes, though this dichotomy oversimplifies the heterogeneity of reactive states (Escartin et al., 2021). In any case, prolonged astrogliosis impairs CNS regeneration (Wilhelmsson et al., 2004). Astrocytes, which tolerate relatively high levels of mitochondrial reactive oxygen species, compensate the limited antioxidant capacity of neurons by producing glutathione for them (Takahashi, 2021; Jimenez-Blasco et al., 2023; Vicente-Gutierrez et al., 2019). Compared with mouse astrocytes, human astrocytes are more susceptible to oxidative stress and less reactive to hypoxic stimuli. However, human astrocytes are more immunologically active, express more defense-response-related genes and secreted cytokines (including IL6 and TL4), and are more likely to exhibit NFkB activation after immunological stimulation (Li et al., 2021a). Reactive astrocytes secrete cytokines and chemokines that regulate BBB function and recruit immune cells to injury sites (Dozio and Sanchez, 2018; Sanmarco et al., 2021).

## 4 Human astrocyte models

### 4.1 Human brain samples

The extent to which available models can represent in vivo human brain function is limited. Furthermore, availability of primary human brain samples, which can be obtained from embryonic or mature postmortem specimens and from surgical preparations, is restricted. Altogether studies of embryonic and postnatal human brain astrocytes have, however, provided information about specific properties of human astrocytes, such as ILAs (Falcone et al., 2021; Aloisi et al., 1992). They have also demonstrated the complexity of intercellular interactions and their disturbances related to diseases such as those found in neurodegenerative conditions (Stringer et al., 2025; Marcadet et al., 2025). Functional study of mature human primary astrocytes can be facilitated by dissociation from tumour tissue and subsequent culture (Hashemi and Hadjighassem, 2017), but such approaches are restricted by the tumour context and require careful timing with sample preparation.

### 4.2 Human iPSC-derived astrocytes

The technology that allowed reprogramming somatic cells back to a pluripotent state and generation of induced pluripotent stem cells (iPSCs) first from mouse and then from human somatic cells (Takahashi et al., 2007; Takahashi and Yamanaka, 2006) has provided new tools to study astrocyte differentiation and function, including their species-specific differences. Given the importance of astrocytes for neuronal function, new technologies have established themselves in this field relatively quickly. One such new technology involves organoids, including both whole-brain organoids and organoids aiming to replicate specific subregions of the human brain. These permit the study of astrocyte biology in a human-specific and physiologically relevant three-dimensional (3D) environment. Brain organoids constitute experimental systems that allow astrocytes to emerge, mature, and interact with neurons, oligodendrocytes and microglia in ways that more closely approximate the *in vivo* human brain than do traditional 2D cultures or animal models. However, even 3D *in vitro* models with hiPSC-derived astrocytes are not able to recapitulate the vast complexity of the human brain due to lack of some important tissue components and limitations in maturation. These limitations are being avoided by analysing human astrocyte function *in vivo* after transplantation of human iPSC-derived cells into the mouse brain. This approach enables monitoring of disease progression and realization of changes induced by the cell transplant in the behavioural phenotype. However, transplantation of cells into the mouse brain microenvironment does not properly account for the human-specific properties of astrocytes, such as the contribution of astrocytes to the maintenance of brain homeostasis.

#### 4.2.1 Astrocyte differentiation in 2D cultures

Several protocols have been validated for differentiation of astrocyte-like cells from human pluripotent stem cells (hPSCs including iPSCs and embryonic stem cells, ESCs). Since astrocytes and neurons are derived from the same progenitors, the initial stages of astrocyte differentiation are directed as in methods used to generate neurons in protocols using neural progenitor cells (NPCs) (Haidet-Phillips et al., 2014; Krencik et al., 2011; McGivern et al., 2013; Shaltouki et al., 2013) (Peteri et al., 2021; Perriot et al., 2021; Oksanen et al., 2017) or oligodendrocyte progenitor cell intermediate (Jiang et al., 2013). The quality of the starting NPC population and the passage number of stocks have been identified to be a critical predictor of successful differentiation of hiPSC-astrocytes (Tcw et al., 2017). The differentiation step into neuroepithelial cells may be combined with patterning (Peteri et al., 2021) and enhanced by inhibiting TGF-β and BMP pathways using small molecules SB431542 and LDN-193189 or Noggin (Krencik et al., 2011; Kumar et al., 2022). The long-lasting culture period >60 days of astrocyte differentiation using NPC intermediate approach is shortened by using commercially available astrocytic growth media (Tcw et al., 2017). A small-molecule inhibitor of the MEK1/2 pathway has also been shown to result in differentiation of astrocytes within weeks by activating STAT1/3 pathway (Garg et al., 2025). In addition, the efficiency of directed differentiation of stable hiPSC-derived neuroepithelial stem cells to astrocytes with a star-shaped phenotype has been shown to be promoted using gelatin coating and higher seeding density, demonstrating the impact of the culture milieu (Voulgaris et al., 2022).

Another main astrocyte differentiation approach uses gene overexpression of transcription factor(s) such as SOX9, NFIA and NFIB. Overexpressing transcription factors in hPSC-derived NPCs are shown to induce rapid and robust astrocyte differentiation within weeks. Direct differentiation using gene overexpression can produce homogenous cultures of astrocytes (Li et al., 2018; Neyrinck et al., 2021; Yeon et al., 2021), whereas methods using NPC intermediate generate more heterogenous astrocyte populations, which may not always be pure astrocyte cultures. Comparison of astrocytes generated using the widely used long, serum-free (LSF) astrocyte differentiation method against a short serum-containing (SS) protocol revealed several differences (Mulica et al., 2023). LSF astrocytes exhibited more pronounced stellate shape with smaller soma and enlarged area of the processes when compared to a fibroblast-like morphology of astrocytes grown with serum. Serum induces astrocytic activation seen in SS astrocytes, but the data suggested that iPSC-derived astrocytes may be activated at baseline. Higher percentage of GFAP+ cells and elevated levels of mature astrocyte marker expression in LSF astrocytes indicated that the LSF protocol produced more mature cells and could be therefore more suitable for initial disease modeling. However, since input from other cell types is required for final astrocyte maturation (Stogsdill et al., 2017), 3D monocultures may promote differentiation further than 2D models (Balasubramanian et al., 2016).

Astrocyte culture characterization is an essential part of astrocyte differentiation process, and it validates the purity and functional state of the produced astrocytes. Immunohistochemistry using astrocyte markers defines cell type and morphology. The common molecular markers are listed in Table 1. Not all of them are specific. GFAP has been found to be a variable marker of astrocyte fate, and furthermore, it is also expressed in human RG. Expression panels of markers and/or global transcriptomic analysis using bulk RNA-Seq or ss/sn-RNA-Seq are recommended to be used for transcript analysis and combined with Western analysis of proteins. Ultimately functional definitions of hiPSC-derived astrocytes are needed. Functional testing includes glutamate uptake analysis and assessment of astrocyte reactivity and cytokine secretion to inflammatory stimuli. Astrocytes communicate via intracellular calcium waves rather than electrical action potentials and capability of astrocytes to respond to external stimuli like ATP or glutamate and to display spontaneous Ca^2+^ transients demonstrate their functionality.

**Table 1:** Common astrocyte markers.

Marker	Expression	Reference
**ALDH1L** aldehyde dehydrogenase 1 family member L1	Marker for immature and mature astrocytes. Localizes in cell body and both proximal and distal processes. Also expressed by RG and adult NSCs.	Forrest et al. (2023)
**AQP4** Aquaporin-4	Marker for mature astrocytes. Localizes in the distal processes and end-feet in vivo and throughout the cell *in vitro*. Also expressed in ependymal cells.	(Forrest et al., 2023; Mayo et al., 2023)
**EAAT1** Excitatory amino acid transporter	Marker for mature astrocytes. Localizes in cell body and distal processes. Also expressed in neuronal processes and oligodendrocytes.	(DeSilva et al., 2012a; Banner et al., 2002; Roberts et al., 2014)
**EAAT2** Excitatory amino acid transporter	Marker for mature astrocytes. Localizes in cell soma and distal processes and endfeet. Expressed also in neurons during early development and oligodendrocytes.	(Forrest et al., 2023; Roberts et al., 2014; DeSilva et al., 2012b)
**GFAP** Glial fibrillary acidic protein	Marker for immature, mature and reactive astrocytes. Localizes in cell body and primary processes, but not in fine processes or endfeet. Expressed in human RG	(Tcw et al., 2017; DeSilva et al., 2012a; Levitt and Rakic, 1980)
**KIR4.1** inwardly rectifying potassium channel	Marker for mature astrocytes. Localization shifts from soma to distal glial processes as development proceeds. Also expressed in oligodendrocytes.	(Higashimori and Sontheimer, 2007; Moroni et al., 2015)
**Nestin**	Marker for immature and reactive astrocytes. Localizes primarily in cell protrusions. Expressed also in NPCs.	(Messam et al., 2000; Thomsen et al., 2013)
**Vimentin**	Marker for immature astrocytes. Localizes in the soma and primary processes, not in distal processes. Also expressed in ependymal cells.	(Schnitzer et al., 1981; O'Leary et al., 2020)
**NF1A** Nuclear factor 1	Marker for both immature and mature astrocytes. Localizes in the nucleus. Also expressed in oligodendrocytes, ependymal cells and a subset of neurons.	(Deneen et al., 2006; Kang et al., 2012)
**SOX9** SRY-Box trans-cription factor 9	Marker for both immature and mature astrocytes. Localizes in the nucleus. Expressed by astrocytes and oligodendrocytes.	Kang et al. (2008)
**S100β** S100 calcium-binding protein β	Marker for mature astrocytes. Localizes in cytoplasm and nucleus. Also expressed in ependymal cells, oligodendrocytes and neurons.	(Tcw et al., 2017; Steiner et al., 2007; Du et al., 2021)

**Table 2:** Summary of special characteristics of human astrocytes.

**Pronounced diversity**	Distinct origins of human cortical astrocytes (Allen et al., 2022b). Human astrocytes display more diverse transcriptomic signatures than mouse astrocytes (Zhang et al., 2016).
**Distinct subtypes of astrocytes**	Interlaminar astrocytes (ILAs) can support a larger brain by modulating neuronal activity and draining excess ions via longer processes (Colombo et al., 1997).
**Morphological differences**	Human astrocytes are larger and structurally more complex than murine astrocytes (Oberheim et al., 2009), to which increased expression of TEAD3 contributes (Ciuba et al., 2025). Human fibrous astrocytes display varicosities while other primates do not (Munger et al., 2022).
**Domain size**	Human astrocytes contact more synapses than mouse astrocytes and exhibit more overlap in domain (Oberheim et al., 2009).
**Blood-brain barrier**	Compared with mice, human astrocytes form specialized structures around blood vessels (Oberheim et al., 2009) and express more AQP4 [88].
**Calcium waves**	Human astrocytes propagate calcium waves faster than rodent astrocytes (Oberheim et al., 2009).
**Ecxitatory signalling**	Human astrocytes acquire glutamate responsiveness later than mouse astrocytes (Zhang et al., 2016). They express more EAAT2 [109] and support excitatory signalling when allografted into mice (Anding et al., 2025).
**Metabolic activity**	Compared to mouse astrocytes, human astrocytes express less metabolism-related genes, they are more susceptible to oxidative stress, and their basal respiration rate is about half of that of mouse astrocytes (Li et al., 2021a). Based on transcriptomic signatures, human astrocytes may be more involved in lipid and neurotransmitter generation (Zintel et al., 2024).
**Inflammatory activity**	Human astrocytes express more secreted cytokines and are more likely to exhibit NFkB activation following immunological stimulation and to activate antigen presenting pathway than mouse astrocytes (Li et al., 2021a).

#### 4.2.2 Astrocytes in organoids

Cerebral organoids are defined as three-dimensional cell culture systems generated from pluripotent stem cells, which self-organize into structures that recapitulate aspects of early human brain development. Important developmental features mimicked by organoids include neural progenitor zone formation, layered organization, and the differentiation of diverse neuronal and glial cell types. Because these models are experimentally accessible, they permit the study of developmental processes and disease mechanisms that are difficult or impossible to directly investigate in the living brain. Organoid models have facilitated the exploration of how astrocytic pathology contributes to disease, including neurodegenerative and neurodevelopmental diseases, as well as how astrocytes influence the vulnerability and resilience of neuronal circuits. To some extent, these models also provide a platform for testing therapeutic strategies aimed at modulating astrocyte function, which suggests that astrocytes, among other neuroglia, might hold an important position in future translational neuroscience.

#### 4.2.3 Human astrocytes in disease modelling

##### 4.2.3.1 Alzheimer's disease

Alzheimer's disease is a good example of how organoid models have furthered our understanding of pathological processes generally, and the role of astrocytes specifically, with respect to these processes. As reviewed in Fernandes et al. (2024), despite technical limitations, such as an absence of vascularization and the fact that the cells constituting the model are physiologically young, unlike in most cases of the real disorder, these models nevertheless recapitulate the hallmark abnormalities of the disease, such as neuronal loss, impaired synaptic activity, and microglial and astroglial activation.

Regarding the role of astrocytes, astrocytic activation has been demonstrated to constitute an early response to amyloid and tau pathology, triggering NF-κB signalling and resulting in the upregulation of pro-inflammatory cytokines (Lian et al., 2015). Single-cell transcriptomic analyses have revealed a dynamic phenotypic change of astrocytes from homeostatic to reactive through an intermediate state in response to their microenvironment conditions (Serrano-Pozo et al., 2024). Astrocyte reactivity contributes to synaptic dysregulation and neuronal stress. Depending on contextual factors, astrocytes may both protect against and amplify neurodegeneration. By recapitulating the heterogeneity of astrocytes, organoid models have allowed the study of APOE4-dependent effects, such as impaired cholesterol transport and exaggerated inflammatory signalling, that may be important drivers of neuronal vulnerability.

##### 4.2.3.2 Autism

Autism represents human-specific disordered behaviour and therefore, human iPSC-derived models can reveal unique mechanisms and are well suited for studies investigating neurobiology of autism. Indeed, astrocytes have been shown to contribute to impaired neuronal function and altered connectivity in autism (Russo et al., 2018). Mixed neural cultures from individuals with non-syndromic autism display reduced synaptic gene expression, diminished glutamate release and neuronal firing rates, and these deficits are exacerbated by astrocytes modelling autism (Russo et al., 2018). Increased reactive oxygen species (ROS), enlarged size, and altered astrocytic IL6 secretion (Russo et al., 2018) are consistent with proposed oxidative stress, inflammation, and immune system dysfunction in the pathogenesis and/or as severity regulator in autism (Pangrazzi et al., 2020). Low expression of complement C4 implies a role for astrocytes in altered synaptic pruning and connectivity seen in autism (Mansur et al., 2021). Furthermore, astrocytes derived from individuals with autism show aberrant Ca^2+^ signalling and induce repetitive behaviour and cognitive deficit when transplanted into the healthy mouse brain (Allen et al., 2022a).

Studies of human iPSC-derived organoids with autism-associated mutations underscore the importance of temporal and cell-type-specific factors in the relation to genetic influence during brain development in the pathophysiology of autism. Autism risk genes have been shown to cause transcriptional changes in neural progenitor lineages and alterations of astrocytes are found in several autism-related organoid models (Brighi et al., 2021; Hong et al., 2023; Li et al., 2025; Astorkia et al., 2024; Dang et al., 2025; Lee et al., 2025; Zillich et al., 2025; Tian et al., 2022; Bury et al., 2024).

##### 4.2.3.3 Epilepsy

Astrocyte involvement in epilepsy is supported by converging evidence from resected human epileptogenic tissue, animal models of epileptogenesis, and reductionist cellular experiments. In mesial temporal lobe epilepsy (MTLE) and other drug-refractory epilepsies, astrocytic glutamate handling is repeatedly implicated (Sarac et al., 2009). A complementary axis involves glutamate–glutamine cycling via astrocytic glutamine synthetase (Eid et al., 2004).

Potassium and water buffering mechanisms provide another well-substantiated astrocyte-to-seizure link (Djukic et al., 2007). Astrocytic Kir4.1 (KCNJ10) underlies spatial K^+^ buffering; astrocyte-specific Kir4.1 deletion in mice produces severe physiological disturbances and seizure phenotypes, and human MTLE hippocampus data show reduced perivascular Kir4.1 immunoreactivity in sclerotic regions—consistent with impaired K^+^ clearance specifically where endfoot specialization would normally stabilize extracellular ion composition (Djukic et al., 2007). AQP4 polarization and perivascular localization interact with K^+^ buffering and glymphatic-like fluid dynamics. In MTLE with hippocampal sclerosis, loss of perivascular AQP4 has been proposed to impair water flux and K^+^ buffering, and AQP4 mislocalization has been documented as a candidate early event in epileptogenesis in experimental models, aligning with the view that astrocyte endfeet dysfunction can be causal rather than purely reactive (Eid et al., 2004).

Inflammatory signalling and reactive astrogliosis represent a mechanistic “multiplexer” rather than a single pathway: astrocytes can both promote and limit seizure development depending on timing, stimulus, and reactive state (Aronica et al., 2012). Furthermore, a distinct, astrocyte-specific “metabolic neuromodulator” pathway in epilepsy is adenosine regulation via astrocytic adenosine kinase (ADK). Astrogliosis-linked ADK upregulation has been proposed as both a biomarker and a driver of seizure aggravation; a primary study demonstrated that astrogliosis in epilepsy can lead to ADK overexpression and seizure worsening, supporting the concept that astrocyte metabolic enzymes shape extracellular neuromodulatory tone (Fedele et al., 2005).

Limitations of non-human epilepsy models are particularly consequential for astrocyte-focused claims. Rodent models reproduce many seizure phenotypes but may not capture human astrocyte size, complexity, and signalling kinetics, and therefore may misrepresent how astrocyte networks coordinate K^+^ buffering, neurotransmitter clearance, and reactive state transitions at human-relevant spatial scales. This mismatch is explicitly raised in comparative astrocyte biology and in disease-specific discussions where human astrocyte differences are framed as a translational bottleneck (Oberheim et al., 2009).

Human brain organoids have addressed epilepsy primarily through genetic epilepsies and lesion-associated epileptogenic disorders where early developmental mechanisms are accessible. The clearest astrocyte-forward organoid evidence in the epilepsy space comes from mTORopathies (notably tuberous sclerosis complex, TSC), where reactive astrocyte features appear early and may precede seizures. A genetically mosaic forebrain organoid model of TSC demonstrated that second-hit TSC2 loss generates transcriptionally activated astrocytes across maturation time points (day ∼120 onward) and argued that glial dysfunction can be an early driver of pathophysiology rather than a secondary response to recurrent seizures (Li et al., 2025).

Independently, patient-derived iPSC astrocytes in TSC show altered proliferation and secretory programs (including enrichment for pathways such as EGF signalling), and astrocyte-conditioned media can shift synaptic balance in neurons; importantly, these synaptic changes were also observed in organoid contexts containing mixed neural and glial populations, supporting the premise that astrocyte-derived factors can non-cell-autonomously bias E/I-relevant synaptic outcomes (Dooves et al., 2021).

Organoid epilepsy modelling nevertheless remains limited for classic astrocyte homeostatic hypotheses (EAAT2/Kir4.1/AQP4/ADK) because many cerebral organoids are developmentally immature, with late-emerging or incompletely polarized astrocytes and frequently absent microglia and vasculature - features that are central to endfoot biology, inflammatory induction regimes, and seizure-like network stabilization. When organoids do exhibit epileptiform activity (e.g., metabolic epilepsy such as GLUT1 deficiency syndrome), the typical readouts emphasize network-level in the multielectrode array (MEA) phenotypes while astrocyte-function assays (glutamate uptake kinetics, K^+^ clearance capacity, endfoot polarity) are less commonly performed, leaving causal attribution to astrocytes underdetermined in many studies (Muller et al., 2024).

##### 4.2.3.4 Parkinson's disease

Astrocytes have been implicated in the pathobiology of Parkinson's disease (PD) through mechanisms that extend beyond “bystander gliosis,” including handling of α-synuclein species, regulation of extracellular glutamate and redox balance, and control of neuronal metabolic resilience. Human tissue and experimental studies support astrocyte involvement in α-syn biology: astrocytes can internalize α-syn aggregates, and aggregated α-syn can propagate between neurons and astrocytes, aligning with models in which astrocytic processing capacity influences proteostatic burden in dopaminergic circuits (Muwanigwa et al., 2024).

A mechanistic theme with direct translational relevance is astrocyte-mediated neurotoxicity in patient-derived systems. In midbrain neuron–astrocyte co-cultures derived from iPSCs, astrocytes carrying PD-associated mutations (e.g., LRRK2 G2019S) have been reported to contribute to neuronal dysfunction through altered mitochondrial state and oxidative stress handling, providing evidence that astrocyte-intrinsic pathology can be sufficient to impair neurons without requiring systemic immune cues (Albert et al., 2022).

Inflammatory reactive-state programs provide a second axis that is increasingly positioned as mechanistically heterogeneous in PD. Broadly, reactive astrocyte states have been proposed to include neurotoxic subtypes induced by microglial cytokine signals; these programs are reported across neurodegenerative contexts including PD, suggesting that glial–immune coupling is an essential interpretive lens for astrocyte findings in synucleinopathies (Liddelow et al., 2017).

Human midbrain organoids have become a particularly influential bridge between PD genetics and astrocyte hypotheses because they allow (i) patterned generation of dopaminergic neurons alongside glia and (ii) longer-term maturation required for proteostasis and aging-like stress to emerge. A major advance is the demonstration that astrocyte proteostasis defects can drive organoid-wide pathology in an early-onset PD model: in DJ-1–deficient iPSC-derived midbrain organoids, impaired lysosomal proteolysis promoted accumulation of advanced glycation end products, increased α-syn phosphorylation and aggregation, and triggered a pro-inflammatory phenotype and reduced metabolic support capacity in astrocytes; co-culture experiments further supported an astrocyte-driven (and astrocyte-rescuable) component of proteolysis deficits in neurons (Parfitt et al., 2024).

Other organoid studies highlight that astrocyte phenotypes can be quantitatively altered by PD mutations in ways that resemble (or are explicitly compared against) human brain samples. For example, PRKN-mutated human brains and iPSC-derived midbrain organoids show reduced astrocytic reactivity and/or altered GFAP^+^ astrocyte representation, supporting a non–cell-autonomous mechanism whereby altered astrocyte responses may condition dopaminergic neuron vulnerability (Kano et al., 2020).

In familial synucleinopathy modelling, SNCA multiplication midbrain organoids recapitulate α-syn pathology with age-dependent aggregation, phosphorylated α-syn species in neurons and glia, and selective dopaminergic neuron loss. These platforms have further linked α-syn pathology to astrocyte senescence-like phenotypes (e.g., nuclear lamina defects and cell-cycle arrest gene induction), elevating “astrosenescence” as a candidate amplifier of dopaminergic degeneration rather than merely a marker of disease stage (Mohamed et al., 2021).

Critical limitations remain. Many PD organoids still lack vasculature and mature neurovascular unit features, meaning astrocyte endfoot polarization, nutrient dynamics, and BBB-like inflammatory gating are modelled incompletely. Microglia can be integrated into midbrain organoids, but protocols vary, and the degree to which microglia–astrocyte interactions reproduce in vivo cytokine milieus (and thus reactive-state transitions) is a key determinant of whether “A1/A2-like” frameworks are testable (Sabate-Soler et al., 2022).

##### 4.2.3.5 Modelling of Multiple Sclerosis

Multiple Sclerosis (MS) has long been framed as an immune-mediated demyelinating disease, yet astrocytes are now strongly supported as early and active constituents of lesion formation, immune cell recruitment, and chronic compartmentalized inflammation. In animal models such as experimental autoimmune encephalomyelitis (EAE), astrocyte NF-κB signaling has been implicated causally: transgenic inhibition of astroglial NF-κB improves outcomes following EAE, directly positioning astrocytes as drivers of neuroinflammatory damage rather than passive responders (Brambilla et al., 2009).

Astrocytes also sit near the core of Th17/IL-17–associated MS immunopathology. In a primary mechanistic study, astrocyte-targeted disruption of IL-17 signaling (via Act1 deficiency) suppressed EAE severity by impairing IL-17–mediated inflammatory gene induction in astrocytes, consistent with a model where astrocytes amplify leukocyte recruitment and lesion expansion through chemokine programs (Kang et al., 2010).

Human genetics and human tissue analyses strengthen this causal framing. A high-impact example is the demonstration that an MS risk variant (rs7665090) enhances NF-κB signaling and astrocyte inflammatory responses in cultured human astrocytes and within MS lesions, linking inherited risk to astrocyte dysfunction and increased CNS access for peripheral immune cells (Ponath et al., 2018).

At the tissue level, modern single-nucleus and spatial-omics studies continue to refine astrocyte heterogeneity within lesion niches. Recent atlases of subcortical MS lesions using paired snRNA-seq and spatial transcriptomics have identified lesion-associated astrocyte subtypes and contextualized astrocyte–myeloid–endothelial communication events at chronic active rims, highlighting that astrocyte states and functions are spatially stratified in ways that 2D systems struggle to reproduce (Lerma-Martin et al., 2024).

Non-human MS models remain indispensable but incomplete. EAE captures key immune drivers but does not fully reproduce MS lesion heterogeneity, chronicity, and human neurodegenerative trajectories. Species differences in astrocyte biology and the absence of human genetic architecture (including risk-variant regulatory contexts) also limit how confidently astrocyte-targeted mechanisms can be translated from rodent to human disease (Brambilla et al., 2009).

Human brain organoids have started to isolate and test glia-intrinsic MS phenotypes in the absence of peripheral immune cells, while also enabling controlled “immune-like” perturbations. A cerebral organoid study spanning multiple MS subtypes (including primary progressive MS) reported dysregulation of the stem cell pool and reduced oligodendrocyte differentiation, with changes in proliferation markers (Ki67/SOX2) and lineage markers and an association with altered p21 expression; by design, this approach probed how patient genetic background can alter CNS lineage dynamics without systemic immune input (Daviaud et al., 2023).

A more explicitly astrocyte- and immune-competent direction is represented by glia-enriched organoid platforms that incorporate microglia and accelerate oligodendrocyte differentiation. In a glia-enriched forebrain organoid system generated via a SOX10-based acceleration strategy, organoids contained neurons, astrocytes, oligodendroglia, and hiPSC-derived microglia and achieved single-cell transcriptional profiles described as similar to adult human brain over ∼8 weeks; exposure to inflamed cerebrospinal fluid (CSF) from MS patients induced macroglia–microglia neurodegenerative phenotypes and intercellular communication signatures aligned with chronic active MS, including rapid oligodendrocyte vulnerability after CSF exposure (Fagiani et al., 2024).

Complementing CSF-driven inflammation models, myelinated organoid systems now allow mechanistic demyelination/remyelination paradigms in a human 3D context. Myelinated human brain organoids with integrated iPSC-derived microglia enabled toxin-induced demyelination followed by spontaneous remyelination, and multi-omics signatures pointed to a central role for microglia in remyelination; notably, pro-remyelinating compounds improved remyelination only in the presence of microglia, underscoring that astrocyte biology in demyelination is likely inseparable from glial–immune interactions in organoid interpretations (Lange et al., 2025).

Even with these advances, MS organoids model some astrocyte-relevant questions better than others. They can productively interrogate (i) glia-intrinsic inflammatory transcriptional states, (ii) astrocyte–microglia signalling loops, and (iii) astrocyte modulation of oligodendrocyte survival and myelin recovery under defined perturbations (CSF, toxins, cytokines). However, they typically lack a fully functional BBB, perfused vasculature, and peripheral immune trafficking—limitations that matter if the manuscript aims to map astrocyte roles in immune entry, lesion initiation at the neurovascular unit, or meningeal/perivascular compartmentalized inflammation (Kistemaker et al., 2025).

##### 4.2.3.6 Schizophrenia

Astrocyte involvement in schizophrenia is supported by converging genetic, postmortem, imaging, and stem-cell evidence, but remains mechanistically heterogeneous and temporally complex because schizophrenia is both highly polygenic and developmentally staged. A central astrocyte-relevant hypothesis concerns glutamatergic dysregulation: astrocytic EAAT1/EAAT2 are primary mediators of glutamate uptake in cortex, and reviews have argued that dysregulation of these transporters could contribute to schizophrenia pathophysiology through altered synaptic glutamate tone and downstream excitotoxic/inflammatory consequences (O'Donovan et al., 2017).

A second mechanistic axis is immune signaling and complement biology. At the population level, complex structural variation and predicted expression of complement component C4 at the MHC locus is strongly associated with schizophrenia risk, and complement-dependent synapse elimination has been incorporated into neurodevelopmental models of schizophrenia. Because astrocytes are major producers and regulators of complement components in the CNS, complement biology naturally implicates astrocyte–microglia–synapse triads even when astrocytes are not the original “risk cell type” (Sekar et al., 2016).

Astrocyte–microglia communication also shapes synapse remodelling through cytokines such as IL-33: astrocyte-derived IL-33 promotes microglial synapse engulfment and circuit development, offering a mechanistic template by which astrocyte immune signals could influence synaptic pruning trajectories relevant to schizophrenia onset windows (Vainchtein et al., 2018).

Human *in vivo* imaging further supports astrocyte involvement at the systems level. A Positron Emission Tomography (PET) study using a MAO-B–binding tracer reported increased signal interpreted as reactive astrocyte involvement in schizophrenia (with correlations to symptom measures), providing an independent line of evidence that astrocyte state changes occur in living patients, not only in postmortem reconstructions (Kim et al., 2024).

Limitations of non-human schizophrenia models are especially salient for astrocyte-centric hypotheses. Rodent models (pharmacologic NMDA antagonism, maternal immune activation, and single-gene perturbations) can reproduce selected behavioral and circuit phenotypes, but they cannot fully reproduce the human genetic architecture (polygenicity; primate-specific gene regulation) or key complement biology nuances (human C4 isotypes relevant to genetic associations). These gaps motivate human iPSC-derived systems as complements rather than replacements for animal studies (Sheridan et al., 2022).

Human brain organoids have addressed schizophrenia predominantly through early neurodevelopmental phenotypes—ventricular zone organization, progenitor survival, neuronal differentiation trajectories, and early circuit formation—while astrocyte functions are less directly interrogated in many studies. In patient-derived cerebral organoids, schizophrenia has been associated with disrupted neurogenesis and progenitor survival, and single-cell analyses emphasize that most cells in control organoids map to neural progenitors or terminal cortical cell types (neurons/glia), whereas schizophrenia organoids can show an increased fraction of non-neuronal “brain-related” cell types at the expense of neurons (Notaras et al., 2022).

A widely cited functional organoid study (using iPSC-derived cerebral organoids from schizophrenia cases and controls) found transcriptomic differences enriched for synaptic biology, neurodevelopment, immune/antigen processing, and mitochondrial pathways, alongside measurable mitochondrial respiration deficits and altered responsiveness in MEA-based stimulation paradigms. These results are biologically compatible with astrocyte metabolic-support and immune-signalling hypotheses, but because the primary readouts were bulk organoid RNA-seq and organoid-level physiological responses, they do not, on their own, assign causality to astrocytes versus neurons without cell-type-resolved perturbations (Kathuria et al., 2020).

More recently, organoid work using defined genetic risk variants has begun to intersect with astrocyte-relevant lineage and maturation questions. Forebrain organoid models carrying NRXN1 heterozygous deletions in both engineered isogenic and schizophrenia patient genetic backgrounds performed longitudinal single-cell transcriptomics from ∼3 weeks to ∼3.5 months and demonstrated developmental timing–dependent effects on neuronal maturation programs; critically for glia framing, the authors note that astrocytes were underrepresented (and thus not quantified) in parts of the dataset, highlighting a common constraint: standard forebrain organoids at these time windows are often at the onset of astrogenesis, limiting statistical power for astrocyte-centred disease claims (Sebastian et al., 2023).

Where schizophrenia models explicitly aim to test immune/complement hypotheses, many groups have used microglia–neuron co-cultures or synaptosome-engulfment assays rather than organoids, precisely because microglia and astrocyte maturity constraints complicate pruning readouts in early organoids. Reviews of patient-derived *in vitro* microglia models emphasize that human cellular systems are required to test complement-associated pruning hypotheses that cannot be faithfully modelled in rodents due to species differences at the C4 locus (Sheridan et al., 2022).

Current schizophrenia organoids appear best positioned to model (i) early developmental programs that precede (or bias) later astrocyte maturation and circuit vulnerability, and (ii) cell-type trajectory shifts that may alter eventual neuron–astrocyte ratios and regional composition. They are less successful at directly modelling mature astrocyte functions such as high-capacity glutamate uptake kinetics (EAAT2-dominant), adult-like endfoot polarity, or fully developed reactive astrogliosis states—unless protocols are extended to longer culture durations, coupled with slicing/vascularization strategies, and/or combined into assembloids with added microglia and vascular components (Qian et al., 2020).

## 5 Conclusions

Astrocytes constitute a significant cell population in brain, performing essential functions. The greater degree of heterogeneity and the morphological, transcriptional, and functional characteristics of human astrocytes compared to murine astrocytes are associated with evolutionary development and higher cognitive abilities in humans. The larger size and more complex structure of human astrocytes compared to murine astrocytes enhance capability to execute versatile functions and multiply bidirectional interactions between astrocytes and neurons. Additionally, human-specific astrocytes, as ILAs with long processes, which mimic the columnar organization of neurons, facilitate connectivity between different cortical layers. The interaction between astrocytes and synapses play crucial roles in modulating synaptic plasticity and behavioural states but also are implicated in the onset and progression of various brain diseases. The advancement of stem cell technology has substantially improved the study of human-specific astrocytes. A deeper understanding of the developmental programs and external factors contributing to the heterogeneity and properties of human astrocytes is essential for elucidation of mechanisms underlying brain disorders and novel treatments. Involvement of astrocytes in the pathophysiology of multiple diseases is evident, as illustrated in the review with disease modelling using human cell-based organoid approaches. The special characteristics of human astrocytes, as summarized in Table 2, raise further questions about regulation of human astrocytes and their potential as therapeutic targets in disease states.

## Contributions

IK, KT, and UKP conducted the primary literature reviews. MC supervised the project, providing guidance on research design and focus. All authors contributed to the writing and final processing of the manuscript.

## Declaration of generative AI and AI-assisted technologies in the writing process

Statement: during the preparation of the final version of the manuscript the authors used GPT-5 (OpenAI) in order to improve fluency and readability. GPT-5 was also used in addition to PubMed to assist in the literature search. After using this tool, the authors reviewed and edited the content as needed and take full responsibility for the content of the publication.

## Declaration of competing interest

The authors declare that they have no known competing financial interests or personal relationships that could have appeared to influence the work reported in this paper.
